# INTERGENERATIONAL RELATIONSHIPS OF RUSSIAN SPEAKERS IN ISRAEL IN THE SHADOW OF PROPAGANDA: FACEBOOK POSTS ANALYSIS

**DOI:** 10.1093/geroni/igac059.3102

**Published:** 2022-12-20

**Authors:** Ksenya Shulyaev, Tova Band-Winterstein

**Affiliations:** University of Haifa, Haifa, Hefa, Israel; University of Haifa, Haifa, Hefa, Israel

## Abstract

Part of the elderly Russian-speaking community in Israel is exposed to the TV channels of the Russian government. These channels are impacted by Russian propaganda and, as such, use false claims and manipulative techniques. The aim of this presentation is to shed light on the impact of Russian media on intergenerational relationships of Russian-speaker families in Israel. We conducted a preliminary qualitative study based on a thematic analysis of Facebook posts (Nf34) from March to June 2022. Analysis revealed four key themes: (1) “Tragedy of many families”, the importance of unity of opinion and the problem of difference, a key element in the emotional well-being (or its lack) of Russian-speaking families at the moment; (2) “It’s useless to explain something”, the perception of older parents who support Putin as victims of “brainwashing”, with comments on “zombies”, totalitarian sects, and psychotropic drugs; (3) “The truth will destroy them”, the perception of disbelief in war as a defensive reaction necessary for the elderly, because the reality may destroy them; (4) “A topic that is not talked about”, a refusal to discuss Ukraine seen as the only way to save the relationship, speaking instead about health issues, daily life issues, and the weather. The findings underline that the Russian-Ukrainian war conflict impacts intergenerational solidarity and closeness, and calls for an intervention that provides support for both the elderly and their adult children.

